# Cutting-Edge Search for Safer Opioid Pain Relief: Retrospective Review of Salvinorin A and Its Analogs

**DOI:** 10.3389/fpsyt.2019.00157

**Published:** 2019-03-27

**Authors:** Jordan K. Zjawiony, Antônio S. Machado, Ricardo Menegatti, Paulo C. Ghedini, Elson A. Costa, Gustavo R. Pedrino, Scott E. Lukas, Octávio L. Franco, Osmar N. Silva, James O. Fajemiroye

**Affiliations:** ^1^Division of Pharmacognosy, Department of BioMolecular Sciences, School of Pharmacy, Research Institute of Pharmaceutical Sciences, University of Mississippi, University, MS, United States; ^2^Laboratory of Medicinal Pharmaceutical Chemistry, Faculty of Pharmacy, Universidade Federal de Goiás, Goiânia, Brazil; ^3^Department of Pharmacology, Institute of Biological Sciences, Universidade Federal de Goiás, Goiânia, Brazil; ^4^Department of Physiology, Universidade Federal de Goiás, Goiânia, Brazil; ^5^McLean Imaging Center, Harvard Medical School, McLean Hospital, Belmont, MA, United States; ^6^S-Inova Biotech, Programa de Pós-Graduação em Biotecnologia, Universidade Católica Dom Bosco, Campo Grande, Brazil; ^7^Centro de Análises Proteômicas e Bioquímicas, Pós-graduação em Ciências Genômicas e Biotecnologia, Universidade Católica de Brasília, Brasília, Brazil; ^8^Programa de Pós-graduação em Patologia Molecular, Universidade de Brasília, Brasília, Brazil; ^9^Centro Universitário de Anápolis, Unievangélica, Anápolis, Brazil

**Keywords:** analgesic, opioid receptors, salvinorin A, side effects, analogs

## Abstract

Over the years, pain has contributed to low life quality, poor health, and economic loss. Opioids are very effective analgesic drugs for treating mild, moderate, or severe pain. Therapeutic application of opioids has been limited by short and long-term side effects. These side effects and opioid-overuse crisis has intensified interest in the search for new molecular targets and drugs. The present review focuses on salvinorin A and its analogs with the aim of exploring their structural and pharmacological profiles as clues for the development of safer analgesics. Ethnopharmacological reports and growing preclinical data have demonstrated the antinociceptive effect of salvinorin A and some of its analogs. The pharmacology of analogs modified at C-2 dominates the literature when compared to the ones from other positions. The distinctive binding affinity of these analogs seems to correlate with their chemical structure and *in vivo* antinociceptive effects. The high susceptibility of salvinorin A to chemical modification makes it an important pharmacological tool for cellular probing and developing analogs with promising analgesic effects. Additional research is still needed to draw reliable conclusions on the therapeutic potential of salvinorin A and its analogs.

## Introduction

Pain management is a challenging medical issue that requires a wide range of expertise and innovative ideas (1). Medicinal chemistry as well as extensive analysis of opioid receptors have increased the possibility of developing novel analgesics that are devoid of detrimental actions (2–6). Pain as an unpleasant sensory and emotional experience has been managed by different classes of drugs such as non-steroidal anti-inflammatory drugs, glucocorticoids, sodium channels inhibitors (local anesthetics), anti-epileptic drugs, tricyclic antidepressants, and opiates (2).

**Figure 1:**
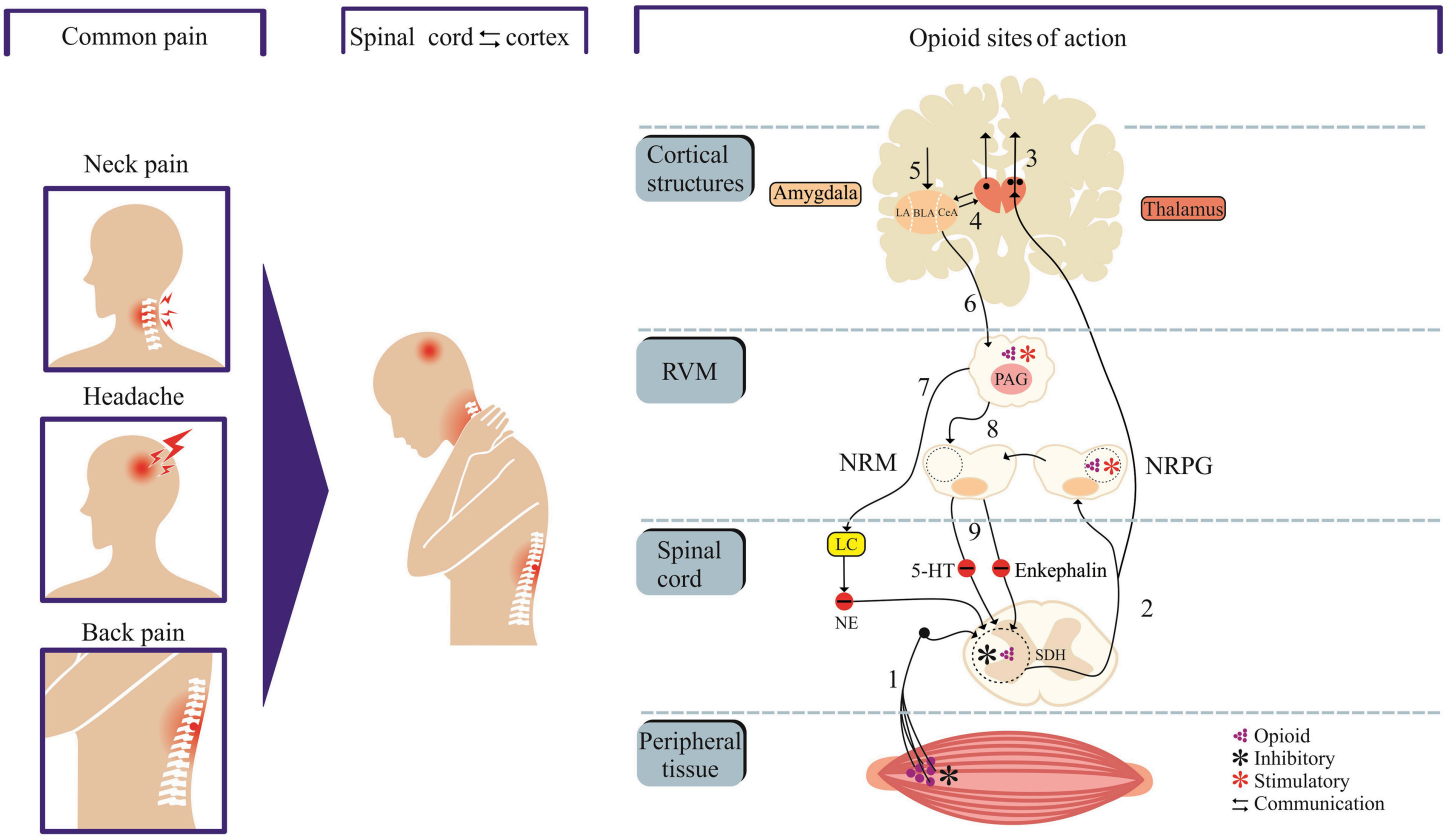
Neural circuits of pain and opioid site of action. Cortex and spinal cord communication modulate pain perception and offer targets for opioid drugs. Neural projections from peripheral tissue transmit nociceptive inputs through primary afferent fibers (1) to the spinal dorsal horn (SDH) before reaching the thalamus (2). Neural projections from thalamus target cortical sites (centers of pain processing, cognition, perceptions and integration) and amygdala (“emotional site”). The amygdala receives nociceptive inputs from the thalamus and cortex. The descending pain control system is mediated through projections from structures such as amygdala and hypothalamus to the periaqueductal gray matter (PAG) which in turn communicates with the rostral ventromedial medulla (RVM). The neural components within RVM [the nucleus raphe magnus (NRM) and nucleus reticularis paragigantocellularis (NRPG)] project to the spinal or medullary dorsal horns to directly or indirectly enhance or attenuate nociceptive transmission. The 5-hydroxytryptamine (5-HT) and enkephalin-containing neurons in the NRM project to the substantia gelatinosa of the dorsal horn and exert an inhibitory influence on transmission. Opioids sites of action include dorsal horn and peripheral terminals of nociceptive afferent neurons where opioids inhibit transmission. Opioids stimulate PAG and NRPG (blue asterisk), which in turn project to the rostroventral medulla. The locus coeruleus (LC) which receives inputs from the PAG releases noradrenalin to the dorsal horn, which in turn inhibits nociceptive transmission. Areas labeled 2–4 in red color and 5–8 in green color represent ascending and descending tracts, respectively.

**Figure 2:**
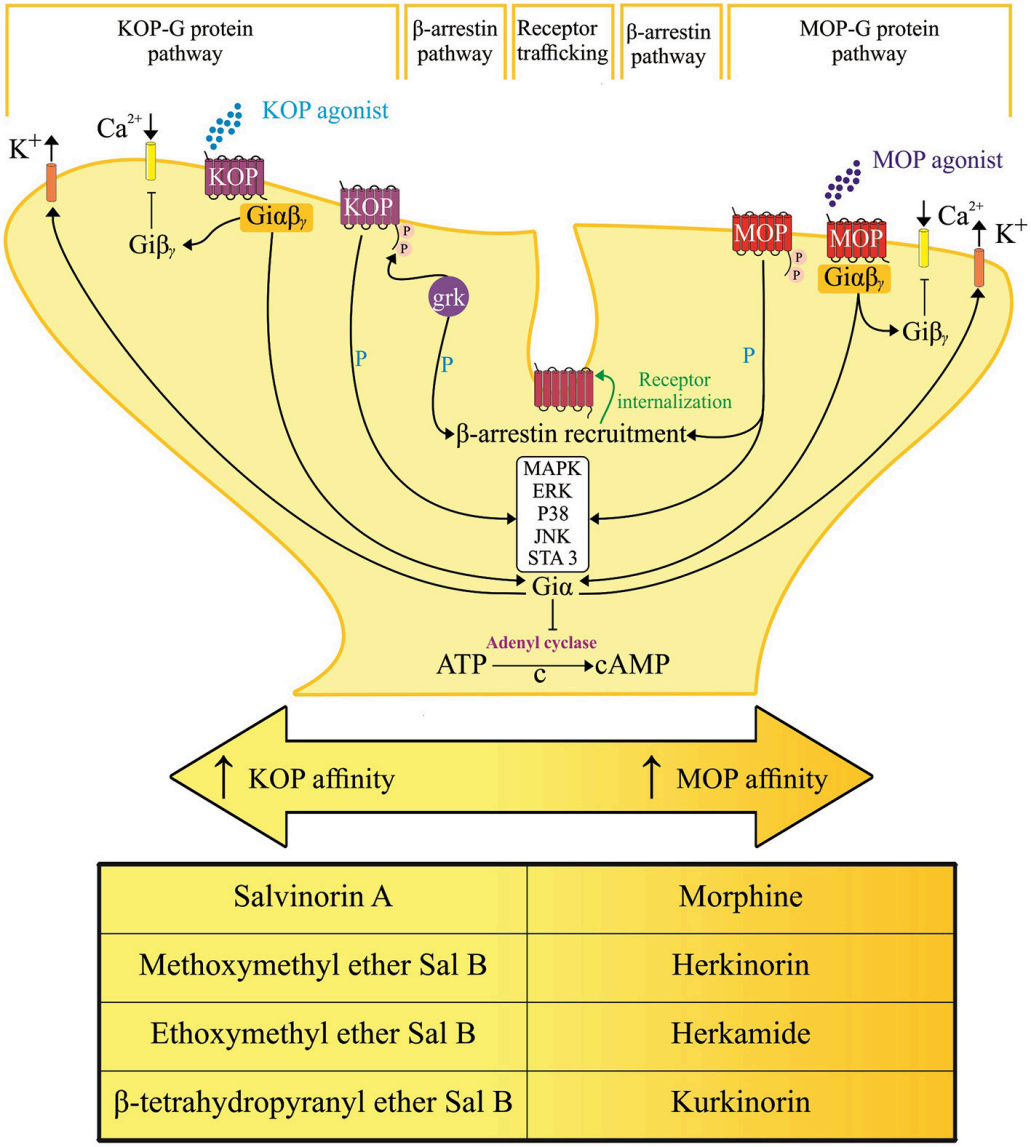
Hypothetical representation of signal transduction and trafficking of mu [μ] and kappa [κ] opioid receptor. Converging downstream pathways are activated by salvinorin A and its analogs with selective action and varying affinity on their respective opioid receptor subtypes. Arrows, activation; T lines, blockade of function; βγ, G protein β-γ subunit; cAMP, cyclic adenosine monophosphate; ERK, extracellular signal-regulated kinase; JNK, c-jun N-terminal kinase; MAPK, mitogen-activated protein kinases; GRK-3, G protein-receptor kinase 3; P, phosphorylation; C → , cyclization of adenosine triphosphate (ATP) into cyclic adenosine monophosphate (cAMP) through the cleavage of pyrophosphate.

Opioid medications that mimic endogenous opioid peptides (dynorphins, endorphins, and enkephalins) typically bind to subtypes of opioid receptors (kappa-KOP, mu-MOP, and/or delta-DOP) to suppress pain (7). In addition, the activation of nociceptin/orphanin FQ peptide receptor (the fourth members of the opioid family of G protein-coupled receptors) by its endogenous peptide nociceptin/orphanin FQ (N/OFQ) modulates stress, reward and pain circuitry in several brain areas (8–11). A schematic representation of the pain and opioid sites of action, as shown in Figure 1, identifies important structures and pain modulatory circuits (12). The detailed account of signal transduction through opioid receptors, as illustrated by Figure 2, has been widely reported (13–17).

The KOP ligands are important research tools and promising molecules for safer treatment of pain (18). The antagonists or partial agonists of KOP could prevent relapse to drug dependence (19–23). The blockade of KOP on dopamine terminals could disinhibit dopamine release in the nucleus accumbens and prevent drug withdrawal-induced dysphoria (24). This receptor remains an important cellular mediator of stress, reward, abuse, emotion, perception (25, 26), sedation (27), hypothermia (28), depression (29, 30), hallucination (31), conditioned place aversion, and locomotion impairment (32). Despite the possibility of undesirable KOP-mediated effects (33, 34), evidence has shown that this receptor subtype is an alternative molecular target for the development of safer analgesics (35).

Recently, Che et al. (3) conducted research on the active-state crystal structure of the KOP complex with a high-affinity agonist to provide molecular details of KOP and overcome the therapeutic limitations of its agonist. In this study, the authors identified residues that are critical for KOP activation and illuminate key molecular determinants of subtype selectivity and signaling bias. The affinity and specificity of drugs to KOP are fundamental to the array of inducible-biological effects (Table 1). The development of drugs that clearly separate pain relief from unwanted side effects has remained challenging and elusive.

**Table 1:** Varying degree of opioid receptor involvement in some pharmacological effect.

**Effects**	**MOP**	**DOP**	**KOP**
Analgesia	+++	±	++
Sedation	++	–	++
Respiratory depression	+++	++	–
Constipation	++	++	+
Euphoria	+++	–	–
Dysphoria	–	–	+++
Depressive behavior	–	–	+++
Hallucination	±	–	+++
Physical dependence	+++	–	+

*MOP, mu-opioid receptor; DOP, delta-opioid receptor; KOP, kappa-opioid receptor; ±, more or less; -, no effect; +, low effect; ++, intermediate effect; +++, high*.

Natural products are important sources of new drugs (36). Several active principles from medicinal plants have been used for pain relief (37). The main active principle of *Salvia divinorum* (38), salvinorin A, had been suggested as a useful research tool toward the development of analgesic drugs (39). Salvinorin A has a distinctive mode of action and pharmacology. Unlike psilocybin and lysergic acid diethylamide (alkaloidal hallucinogens which interact with specific serotonin receptor subtypes), the hallucinogenic effect of salvinorin A has been associated with its potent and selective KOP agonism. Salvinorin A shows no significant binding to over 50 other pharmacologically important receptors, transporter proteins and ion channels (40). As a non-nitrogenous KOP agonist (40), this compound differs from typical alkaloid opioid agonists. Recently, the potent antinociceptive effect of salvinorin A was reported in the neuropathic pain model (41). Over the years, medicinal chemists have synthesized several hundred analogs of this compound including herkinorin, kurkinorin, P-37, PR-38, methoxymethyl- and ethoxymethyl ether of salvinorin B, and β-tetrahydropyranyl ether of salvinorin B (4, 6, 42). Previous preclinical reports on salvinorin A and its analogs have revealed their promising antinociceptive effects (41, 43, 44). Hence, this review explored the structural and pharmacological profiles of salvinorin A and its analogs toward the development of new analgesic drugs.

## Salvinorin A and its Analogs: Structure-Activity Relationship

### Salvinorin A

**Figure 3:**
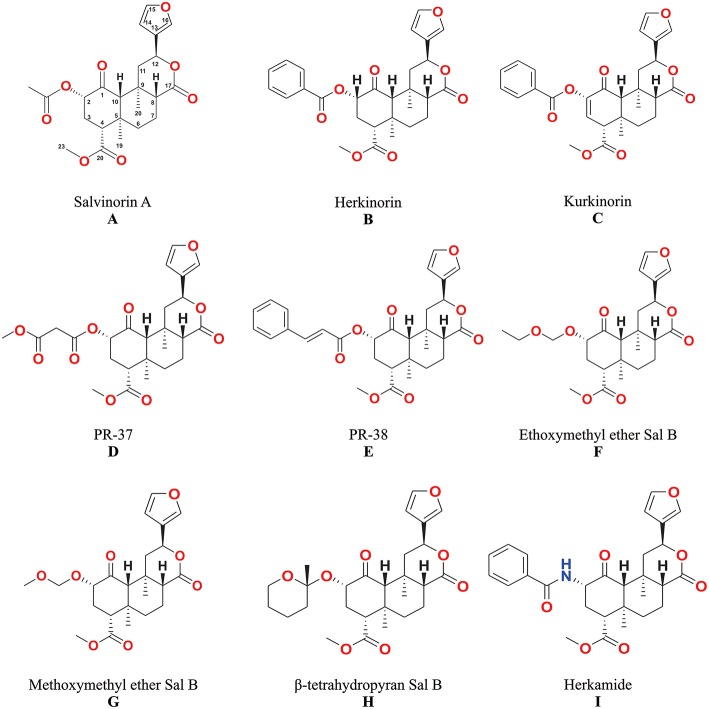
The structures of salvinorin A **(A)** and its analogs **(B–I)**.

The unique biological effects of salvinorin A (Figure 3A) have motivated many scientists to seek correlations between its chemical structure and pharmacological activity (4, 6, 42).

Following the determination of the salvinorin A structure through A single-crystal x-ray analysis [(38, 45), molecular modeling studies were performed to determine the interaction of this compound with KOP (40). Initially, the salvinorin A crystal structure (45) was docked by superimposition of its aromatic centroids and the carbonyl atoms with those of bound U69593 (known KOP agonist which shares structural similarity such as an aromatic ring and ester carbonyl groups separated by a short bond with salvinorin A). As a hydrogen bond acceptor, the carbonyl functionality supports the proposed role of Y139 and its interaction with the lactone carbonyl of salvinorin A (40, 46).

Previous study showed the list of residues that could form the salvinorin A-binding site of the KOP (40). The KOP models could accommodate the furan oxygen and 4-methoxycarbonyl functionality but not the 2-acetoxy group (40). The key residues in KOP that are responsible for the high binding affinity and efficacy of salvinorin A as well as important contacts between this compound and KOP have been identified through mutagenesis studies (6, 47). Potent and efficacious interactions of this compound with KOP are due to novel binding modes within a common three-dimensional space for binding and activating KOP (47).

Additional studies correlated the structure and activities of salvinorin A with the potential binding site on KOP. For instance, a change to the furan ring resulted in analogs that are more sterically demanding than a one-for-one aryl ring replacement (6). Sterically hindered environment of C-1 carbonyl of salvinorin A is not essential for activity as it is incapable of forming specific donor/acceptor contacts with residues in the receptor model, (48). In contrary, the 2-acetoxy group of salvinorin A which makes specific donor/acceptor contacts in the model is required for activity (45). Meanwhile, the lack of consensus binding model makes generalization of structure-activity relationships a challenge (6).

According to Yan (47), salvinorin A uses its flexible functional groups at C-2, C-4 and C-12 to optimize KOP interactions and stabilize itself in the binding site. Moreso, this compound also takes advantage of the conformational changes induced by G protein-coupling to facilitate active state stabilization and activation of downstream signaling events.

Salvinorin A, a neo-clerodane diterpenoid with seven stereogenic centers and three different types of ester functionalities, is a challenging substrate for chemical modifications because minor modifications can result in a complete loss or increase in pharmacological activity. For example, a product of hydrolysis of the C2 acetoxy side chain (salvinorin B) is totally devoid of activity, but other changes to this position actually demonstrate the highest KOP binding affinities (4, 6). Structural modifications of salvinorin A at the C1, C4, C12, and the C17-positions have been mostly associated with a reduction in KOP binding affinity (4, 6, 49). Over the years, the carbonate, carbamate, ester, ether, amine, amide, sulfonic ester, sulfonamide, thioester, halide, and other groups have been introduced to the salvinorin A molecule with a wide variety of outcomes (6, 50).

Furthermore, potential interactions of salvinorin A with other receptors have been either hypothesized or demonstrated preclinically by some researchers. Previous study showed that salvinorin A allosterically modulates MOP binding (51). The *in vitro* testing which showed the binding affinity of salvinorin A (EC50 values of 89 nM) against the D2 High receptor and blockade by 10 μM S-sulpiride (an antagonist of DRD2) has resulted into the discussion of partial agonism of salvinorin A at D2 receptor (52). Moreso, computational studies have predicted CB1, CB2, or DRD2 as a potential targets of salvinorin A (53). In an *in vivo* test, the attenuation of neuropathic pain by Salvinorin A was blocked by CB1 and KOP antagonists (41). The inhibition of the effects of salvinorin A on colonic motility by antagonists of OPRK, CB1 and CB2 *in vitro* and largely by antagonists of OPRK *in vivo* (54) suggests mechanistic complexity in the activity of salvinorin A as against widely acclaimed KOP selectivity.

## Analogs From the Modification of Salvinorin A at C-2

### Herkinorin

Analogs with bulky alkyl esters at C-2 resulted in a loss in affinity for KOP (55), but the replacement of the alkyl with aryl esters at C-2 results in a lower affinity and potency for KOP; and an increase in affinity for MOP (56). Herkinorin (Figure 3B) is the first example of salvinorin A derivative with Ki at MOP (12 ± 1.0 nM) while still retaining lower affinity at KOP (90 ± 2.0 nM).

### Methoxymethyl and Ethoxymethyl Ether of Salvinorin B

These compounds have an alkoxyalkyl ether bond, which replaced acetoxy group at C-2. The alkoxy methyl ether substituents improved KOP affinity and potency. The methoxymethyl ether of salvinorin B (Figure 3G) has higher binding affinity to KOP (Ki = 0.60 ± 0.1 nM) and potency (EC_50_ = 0.40 ± 0.04 nM) than salvinorin A. The putative synergistic binding interactions of the additional oxygen in the substituent have been associated with the higher affinity and potency (57, 58). The ethoxymethyl ether of salvinorin B (Figure 3F) also displayed a higher KOP binding affinity (Ki = 0.32 nM) and potency (EC50 = 0.14 nM) than other salvinorin A analogs (4, 59).

### Methyl Salvinorin B-2-O-Malonate and 2-O-Cinnamoylsalvinorin B

Previous studies have shown the synthesis and biological activities of Michael acceptor-type of salvinorin A analogs, such as methyl salvinorin B-2-*O*-malonate (PR-37) and 2-*O*-cinnamoylsalvinorin B (PR-38) (42, 54, 60). The addition of a second H-binding acceptor leads to the development of a malonate analog (PR-37) (Figure 3D) that displayed a 3-fold improvement in KOP affinity (Ki = 2.0 ± 0.9 nM) (42). However, other malonic ester substitutions with different carbonyl spacings reduced biological activity (6). The replacement of the acetate substituent with the spirolactone group caused a restriction in bond rotation and a decrease in potency (61). The analog with cinnamic ester functionality (PR-38) (Figure 3E) displayed not only KOP affinity (Ki = 9.6 ± 2.0 nM) but also MOP (Ki = 52 ± 9.0 nM) with 5.4 MOP/KOP selectivity (42).

### Herkamide

Tidgewell (62) showed a lower KOP affinity as a result of bioisosteric exchange of the 2-acetoxy subunit of salvinorin A (Ki = 1.9 ± 0.2) with acetamide (Ki = 30 ± 2.0 nM). Although the introduction of a phenyl ring in the herkamide analog (Figure 3I) decreased KOP affinity, an increase in affinity for MOP - Ki = 3.1 ± 0.4 as compared to herkinorin Ki = 12 ± 1.0 nM was reported. In addition, herkamide MOP selectivity (KOP/MOP = 0.0004) was shown to be higher than that of herkinorin (KOP/MOP = 0.13).

### Kurkinorin

The introduction of a double bond between C-2 and C-3 in herkinorin resulted in the new analog kurkinorin (Figure 3C). *In vitro* functional assay revealed that kurkinorin was more selective for MOP (>8,000-fold selectivity over KOP) than morphine (66-fold selectivity over KOP) and herkinorin (4.25-fold selectivity over KOP). Moreover, kurkinorin has similar potency when compared to MOP agonist such as DAMGO in forskolin-induced cAMP accumulation assays (63).

### β-Tetrahydropyranyl Ether of Salvinorin B

The relative flexibility of the acetoxy (C-2) subunit and potential adoption of different conformations when interacting with KOP has been hypothesized and studied in β-tetrahydropyranyl ether of salvinorin B (Figure 3H). Prevatt-Smith et al. (59) applied the concept of conformational restriction toward the development of ligands as tools to elucidate KOP affinity and potency. The new analog β-tetrahydropyranyl ether of salvinorin B showed slightly higher KOP affinity (Ki = 6.21 ± 0.4) than salvinorin A (Ki = 7.40 ± 0.4). This result showed that the rotational restriction strategy as proposed by Prevatt-Smith et al. (59) only led to small changes in binding values.

## Analogs From the Modification of Salvinorin A at C-4

Unlike the modification of salvinorin A at C-2, the structural modifications at the C-4 present challenges because selective hydrolysis of methyl ester requires a more drastic condition that often leads to C-8 epimerization (6). Hence, additional efforts are often needed toward the separation of diastereoisomers. This seems to be part of the reason why analogs from C-4 position are fewer than that of C-2. A total loss of KOP binding affinity (Ki > 1000 nM) was reported for methyl, propyl and methoxymethyl esters at C-4 (57, 64).

The loss of KOP binding affinity in the long-chain ester has been associated with the fact that the pocket where the methyl ester fits is small and delimited by Trp287 and Tyr320 amino acids residues (65). Reduction of ester to alcohol has also led to an 87-fold decrease in KOP binding affinity as compared to salvinorin A (58). Consistent with these data, other studies have shown 33-fold and 385-fold losses in KOP affinity (64, 66). Some esters with modified regiochemistry with the exception of cyclopropyl ester showed a 17-fold loss of affinity (64).

The replacement of methyl ester at C-4 with amides or amines resulted in a 535-fold loss or a total loss (Ki > 10,000 nM) of KOP binding affinity, respectively (66). With the exception of alanine, the introduction of amino acid at C-4 resulted in a total loss of affinity (57, 64). Some substitutions with functional groups, such as carboxylic acid and aldehyde, have also resulted in total loss of KOP binding affinity (64).

## Analogs From the Modification of Salvinorin A at C-12

The study of the analogs with substitutions at C-12 (the furan ring of salvinorin A) has attracted attention as a result of their metabolic stability (67). Although the interaction between furan and KOP is not well-established, the removal of this ring in salvinorin A resulted in total loss of activity while its hydrogenation resulted in a 7-fold loss (Ki = 14 ± 1.0 vs. 1.9 ± 0.2) in KOP binding affinity (68). Perhaps the possibility of hydrogen bonding, hydrophobic interactions or even π-π stacking type is essential for a receptor's recognition. (69), the regiochemical modification of the furan resulted in a 2-fold decrease in potency (EC_50_ = 12.2 ± 4.4 nM) without significant change in efficacy (E_max_ = 97 ± 2%) when compared to salvinorin A (vs. EC_50_ = 6.11 ± 0.04 nM and vs. E_max_ = 97 ± 8%).

## Discussion on Antinociceptive Effect of Salvinorin A and Its Analogs

Medicinal chemists have consistently modified salvinorin A structures to produce a wide range of analogs (4, 6). These efforts have helped to further understanding of salvinorin A chemistry and pharmacology as well as developing new compounds with potential therapeutic values (70). Since the identification of salvinorin A by Bücheler et al. (71), scientists have shown interests in its analgesic potential. Recently, the effectiveness of salvinorin A in a rodent model of pain showed that this compound could be beneficial for neuropathic pain relief (41). Salvinorin A is lipophilic, and it is mainly absorbed through the respiratory tract and to a lesser extent by the oral mucosa (72). Following the isolation and characterization of salvinorin A *in vitro* (receptor binding and functional assays) and *in vivo*, chemical modification of this compound led to changes in pharmacological parameters including stability, bioavailability, binding affinity, potency, functional activities, and selectivity (6, 73, 74). Hence, the activities of salvinorin A and its analogs offer clues toward the development of safer analgesics.

The pharmacological characterization of salvinorin A has been widely published (75). Salvinorin A was reported as a selective agonist of KOP through a competitive radioligand binding affinity assay (40). This pharmacological profile was subsequently replicated and confirmed by the findings of Chavkin and co-collaborators (76). As an important pharmacological target, the KOP has been implicated in the antinociceptive effect of salvinorin A (28, 77–79). Consistent with these reports, the potent antineuropathic pain of salvinorin A was blocked by the administration of a KOP antagonist (41). In addition to its selectivity to KOP, salvinorin A has a very high KOP potency. For instance, doses as low as 200 micrograms of this compound produce hallucination (31).

The pharmacology of salvinorin A is considered unique as a result of its structure and binding to the KOP (5). Despite being a potent activator of KOP-mediated G protein signaling, receptor internalization by salvinorin A is still poorly known (79). The internalization of receptor and β-arrestin recruitment are two cellular events that often accompany G protein activation (5). These cellular events have been linked to the underlying mechanism of unwanted side effects (80, 81). Hence, specific functional groups or structural features of salvinorin A that are critical to KOP interaction could be explored to repurpose analogs with only antinociceptive effect.

As highlighted above, binding affinity parameter has consistently been used for preclinical screening and as a basis for structural activity relationship studies. However, varying values of binding affinity data of salvinorin A and its analogs from different laboratories have raised questions about their reliability. Inconsistent data or lack of replicability of binding data could have resulted from the use of different radioligands to measure binding constants for the same analog (6).

Nature provides important chemical and pharmacological clues through the hydrolysis of salvinorin A at C-2 position that leads to salvinorin B and eliminates KOP activity (4, 48, 76). In this manner, the C-2 position represents a critical pharmacophore for salvinorin A and KOP interaction (57, 66, 76). In addition, the therapeutic potential of salvinorin A is limited as a result of its fast hydrolysis at the C-2 position by esterases (76, 82). Previous study which showed loss of the antinociceptive activity of salvinorin A after 20 min of intrathecal injection confirmed its short duration of action (77).

Medicinal chemists have advanced understanding of salvinorin A through its analogs (4, 6, 42). As mentioned earlier in this review, the aromatic substitution through the introduction of a phenyl group at C-2 as in herkinorin reduced KOP and increased MOP binding affinity (56). The structural change and selective activation of opioid receptors seem to be important clues to the antinociceptive effect of this compound. However, there is no data to exclude the possible hallucinations and physical dependence that are often associated with KOP and MOP agonists, respectively. Selective activation of opioid receptor-mediated beneficial pathways over deleterious signaling pathways offers an alternative therapeutic opportunity (3, 83, 84). According to some authors, the selective activation of Gi/o protein-mediated pathways over arrestin-mediated signaling could be a clue to designing safer drugs (85–87).

Some experimental data on salvinorin A analogs have shown preferential activation of G protein, β-arrestin recruitment among other molecular targets. Previous data showed that herkinorin promoted phosphorylation of MAP kinases ERK1/2 independent from β-arrestin-2 signaling and without promoting MOP recruitment of β-arrestin-2 (88). The β-arrestin-2 knockout mice with opioid treatment exhibited reduced opioid tolerance, improved the antinociceptive effect devoid of respiratory depression and constipation (89–91). Some authors have associated opioid dependence with the internalization of G protein—coupled receptors (80, 81, 92). The fact that herkinorin did not promote MOP internalization makes its potential application as an analgesic far more interesting.

Kurkinorin, which is considered to be extremely selective to MOP, showed a complete pharmacological change from salvinorin A which is known for a very high KOP binding affinity (63, 76). Moreover, kurkinorin also has greater selectivity for MOP than herkinorin. However, kurkinorin was found to recruit β-arrestin 2 (EC50 > 140 nM) with an efficacy of 96% and a bias factor of 0.57 when compared to DAMGO (63). Although these data suggest that kurkinorin may produce a morphine-like antinociceptive effect, chemical changes in the structure of these compounds provide important information on the molecular features that are necessary for molecular recognition of a ligand by opioid receptors. Hence, additional modification could be sufficient to prevent potential undesirable activity of kurkinorin without compromising antinociceptive property.

Animal models of abdominal pain and pruritus have also been explored to further the study on some salvinorin A analogs and their potential antinociceptive effect (43, 93). The aromatic analogs such as PR-37 and PR-38, which displayed lower affinity for KOP, blocked nociceptive responses. The intraperitoneal administration of PR-38 (10 mg/kg) and salvinorin A (3 mg/kg) elicited a significant decrease in pain-related behaviors. The higher dose of this analog suggests that salvinorin A is more potent than PR-38. In 2015, Salaga et al. showed attenuation of compound 48/80-induced itch responses in mice by PR-37 and PR-38 (93). The antiscratch activity of PR-37 was blocked by the selective nor-binaltorphimine (KOP antagonist), and that of PR-38 by β-funaltrexamine (selective MOP antagonist). In this study, both PR-37 and PR-38 induced antiscratch activity at the same doses of 10 and 20 mg/kg.

Pharmacological evaluation of β-tetrahydropyranyl ether of salvinorin B has provided effective insight into the antinociceptive activity of this analog and salvinorin A. The non-linear regression analysis of hot water tail-withdrawal latency revealed β-tetrahydropyranyl ether of salvinorin B to be more potent (ED_50_ 1.4 mg/kg) than salvinorin A (ED_50_ 2.1 mg/kg) (44). In addition, salvinorin A and β-tetrahydropyranyl ether of salvinorin B reduced both phase 1 nociceptive pain and phase 2 inflammatory pain in formalin test. The β-tetrahydropyranyl ether of salvinorin B produced a longer duration of action in the tail-withdrawal assay when compared to the salvinorin A. An increased duration of action has been attributed to the substitutions of tetrahydropyran group at C-2 position (44).

## Clinical and Ethnopharmacological Considerations of Salvinorin A and Its Analogs

Currently, except for the ethnopharmacological reports, there is a dearth of clinical data to support the analgesic property of *S. divinorum* and salvinorin A (94). Salvinorin A has a long history of use as an entheogen by the shamans/healers of the Mazatec people (95). The ingestion of this plant species induces a short-lived inebriant state with intense, bizarre feelings of depersonalization (71, 96). At low infusion doses, the plant leaves have been used to treat headache, rheumatism, anemia, constipation, anuria, and diarrhea (97, 98). These pharmacological effects among others have been attributed to salvinorin A (99).

Headache is a daily painful experience that affects individuals of all ages (100, 101). Medicinal plant application to ameliorate unpleasant sensory and emotional experience that is associated with a headache is a common practice. The analgesic effect of *S. divinorum* leaf through infusion, chewing and swallowing could be attributed to its salvinorin A content. One kilogram of dried leaves or eight kilograms of fresh leaves of *S. divinorum* delivers about 1.5 g of salvinorin A when smoked, vaporized and inhaled (31, 99).

In addition to the antiheadache property of salvinorin A, its potential role in the antirheumatoid activity of *S. divinorum* leaf preparation further supports the antinociceptive property. The origins of rheumatoid arthritis (RA) remains controversial, and its origins in the New or Old World are subjects of several scientific works (102). However, several studies have shown higher prevalence of RA among the natives and women between the ages of 35 and 50 (103, 104). The treatment of RA among the Mazatec peoples is expected given its high incidence. The reports of the use of *S. divinorum* for RA treatment are plausible, in view of the potent antinociceptive effect of salvinorin A against chronic and neuropathic pain (41).

The effect of *S. divinorum* infusion against anemia, constipation, anuria, and diarrhea could provide additional therapeutic benefits not only from its main isolate but also from some of salvinorin A analogs (105, 106). Gastrointestinal tract discomfort, constipation and diarrhea are among the therapeutic limitations of some analgesic medications including NSAIDs and opioids (107).

## Addictive Properties: Comparison of Opioids With Salvinorin A and Its Analogs

Salvinorin A as a selective KOP agonist does not elicit an addictive effect. This property has stimulated research into its semi-synthetic analogs as therapeutic agents (108). The activation of KOP produces anti-addictive effects by regulating dopamine levels in the brain (106). Unlike salvinorin A, there is dearth of scientific data on the addictive or anti-addictive tendency of its analogs. This may be connected with the fact that most of these compounds were not studied *in vivo*. However, based on the neurobiology of addiction, analogs with high affinity for MOP including kurkinorin, herkamide, and herkinorin need to be evaluated for addictive property and compared with the available drugs being used to manage pain. In addition, the potential addictive property of analogs with high KOP affinity including methoxymethyl ether of salvinorin B, β-tetrahydropyranyl ether of salvinorin B, and ethoxymethyl ether of salvinorin B needs to be evaluated since KOP often promote aversion, withdrawal and abstinence (109). There are possibilities of analog such as 2-*O*-cinnamoylsalvinorin B with moderate dual MOP/KOP agonism to retain analgesic effect without addiction. However, biased activation of different signaling pathways that are associated with KOP is key to non-addictive, addictive, or anti-addictive effect (106). According to (109), both MOP and KOP contribute to specific aspects of addiction by triggering its onset and progression.

The reports on the side effects of salvinorin A such as locomotor decreases aversion, anhedonia, memory impairment, depressant-like behaviors, hallucinations among others (73, 106, 110) may have negatively reduced its therapeutic values. In the previous report, the intraperitoneal administration of salvinorin A significantly lowered dopamine levels in the caudate putamen to elicit conditioned place aversion in rodents (32). Salvinorin A-induced potentiation of dopamine re-uptake transporter function has been reported as a plausible mechanism of the decreases in dopamine levels (106). The neurobiology of salvinorin A induced memory impairment and other side effects is still unclear. Although there are no established structural activity relationships in respect of these side effects, the analogs of salvinorin A still hold promise for the future development of analgesic drugs without addictive and other side effects. Hence, robust preclinical studies and clinical trials will ultimately reveal the therapeutic potential of these analogs.

## Final Considerations and Conclusions

Salvinorin A was the first non-nitrogenous opioid receptor agonist. Non-nitrogenous nature of this compound can be attributed to its unique biological activities. According to Cunningham et al. (4), non-nitrogenous or non-alkaloids are promising scaffolds for new drug development. Despite the reports on the opioid receptor mediated antinociceptive effect of salvinorin A, its instability, short duration of action and side effects remains sources of concern. Systematical modification has increased understanding of the important role of substitutions at different positions of the salvinorin A scaffold and increased the possibility of developing safer analgesic drugs. Currently, the data on the binding affinity of salvinorin A analogs are yet to be correlated with possible low side effects and therapeutic advantage over existing drugs. As ligands can bind well without stabilizing the receptor's active conformation, lower or higher binding affinity is not synonymous with efficacy and potency.

Renewed focus on molecular targets seems to be promising because the activation of KOP or MOP could selectively affect β-arrestin or G-protein signaling. As the arrestin signaling pathway is responsible for many adverse effects of opioids, biased agonism for the G-protein pathway could retain analgesic effects with a reduced side effect. Hence, additional research efforts are still needed toward: (i) the modification of salvinorin A, (ii) comprehensive study of opioid receptors and associated molecular targets, (iii) extensive *in vivo* assays of salvinorin A analogs, iv. optimization of structural and pharmacological clues to develop safer analgesics.

On a final note, it is clear that FDA approval of salvinorin A as an analgesic constitutes an uphill task, however, the body of work reviewed here shows that some analogs of salvinorin A could translate to valuable drugs for the management of pain.

## Author Contributions

JF and JZ conceived the presented idea. JF, AM, PG, EC, GP, and OS wrote the manuscript with support from JZ, SL, RM, and OF. All authors discussed the content and contributed to the final manuscript.

### Conflict of Interest Statement

The authors declare that the research was conducted in the absence of any commercial or financial relationships that could be construed as a potential conflict of interest.
